# Metagenomic analysis of *Raphidiopsisraciborskii* microbiome: beyond the individual

**DOI:** 10.3897/BDJ.9.e72514

**Published:** 2021-10-21

**Authors:** Paula Vico, Andrés Iriarte, Sylvia Bonilla, Claudia Piccini

**Affiliations:** 1 Instituto de Investigaciones Biológicas Clemente Estable, Montevideo, Uruguay Instituto de Investigaciones Biológicas Clemente Estable Montevideo Uruguay; 2 Instituto de Higiene, Facultad de Medicina, UDELAR, Montevideo, Uruguay Instituto de Higiene, Facultad de Medicina, UDELAR Montevideo Uruguay; 3 Sección Limnología. Facultad de Ciencias, UDELAR, Montevideo, Uruguay Sección Limnología. Facultad de Ciencias, UDELAR Montevideo Uruguay

**Keywords:** *Raphidiopsisraciborskii*, microbiome, 16S rRNA metagenomics, phycosphere

## Abstract

*Raphidiopsisraciborskii* is a toxic, invasive bacteria with a defined biogeographic pattern attributed to the generation of ecotypes subjected to local environmental filters and to phenotypic plasticity. The interactions taking place between the cyanobacterium and the other bacteria inhabiting the external polysaccharide-rich matrix surrounding the cells, or phycosphere, may be ecotype-specific and would have different influence on the carbon and nutrient cycling in the ecosystem. Here, we describe the bacterial community or microbiome (assessed by 16S rRNA metagenomics) associated to two *R.raciborskii* strains that have been described as different ecotypes: the saxitoxin-producer MVCC19 and the non-toxic LB2897. Our results showed that both ecotypes share 50% of their microbiomes and differ in their dominant taxa. The taxon having the highest abundance in the microbiome of MVCC19 was *Neorhizobium* (22.5% relative abundance), while the dominant taxon in LB2897 was the Planctomycetes *SM1A02* (26.2% relative abundance). These groups exhibit different metabolic capabilities regarding nitrogen acquisition (symbiotic nitrogen-fixing in *Neorhizobium* vs. anammox in *SM1A02*), suggesting the existence of ecotype-specific microbiomes that play a relevant role in cyanobacterial niche-adaptation. In addition, as saxitoxin and analogues are nitrogen-rich (7 atoms per molecule), we hypothesise that saxitoxin-producing *R.raciborskii* benefits from external sources of nitrogen provided by the microbiome bacteria. Based on these findings, we propose that the mechanisms involved in the assembly of the cyanobacterial microbiome community are ecotype-dependent.

## Introduction

Cyanobacteria have a polysaccharide-rich microzone outside the cell wall that surrounds the cells, filaments or colonies, which are colonised with heterotrophic bacteria. Despite the recognised role of heterotrophic bacteria in carbon and nutrient recycling of aquatic ecosystems, little is known about the composition and the interaction of these bacteria with the bacteria. In this microzone, called the phycosphere (Bell and Mitchel 1972), the metabolites are readily exchanged before their diffusion to the water and represent a central meeting place for bacteria and bacteria (Seymour et al. 2017). The interactions occurring between individual organisms within this phycosphere have an ecosystem-level effect on several processes, for example, carbon and nutrient cycling, toxin biosynthesis etc. The incorporation of bacteria into the phycosphere likely occurs through chemotaxis, random contacts and vertical transmission (Seymour et al. 2017).

The close association between bacteria and bacteria that occurs in the phycosphere may be a strategy to achieve a higher number of genes and functions to cope and thrive through a range of environmental conditions (Humbert et al. 2013). These interactions translate into a selective advantage for both partners, which widen the spectrum of “goods” that they can obtain. The concept of the “common good” proposes that natural selection would keep those functions available in the whole community (Morris et al. 2011). By sharing these metabolites that are extracellularly available, the ability to synthesise them by themselves would no longer be necessary (Pande and Kost 2017). Thus, the bacterial species that are able to co-evolve in synergy and interaction would constitute the microbiome of a community or the "interactome" (Cook et al. 2019). This has been addressed in the cyanobacterium *Microcystis* sp., where a metageneomics-based study of blooms from twelve lakes showed that their microbiomes share a large number of functional genes despite the fact that bacteria were taxonomically distinct at the 16S rRNA level (Jankowiak and Gobler 2020). As a consequence, changes at the taxonomic level would be functionally equivalent and guarantee the permanence of essential metabolic functions.

The synthesis of the external polysaccharide-rich (EPS) matrix that surrounds the cyanobacterial cells is thought to be a physiological response to fluctuations in environmental conditions, allowing bacteria to maintain their fitness and also the associated microbiota (Rossi and De Philippis 2015). The chemical characteristics of EPS and its abundance depend on the species and strain of bacteria and the culture or environmental conditions. Under oligotrophic conditions, EPS is the source of available organic carbon for heterotrophs, while during blooms, the relative abundance of bacteria living in the EPS varies depending on the biomass of the bacteria and its EPS composition (Woodhouse et al. 2018). It is then possible that bacteria having different metabolic functions are selected and that structural and functional differences in the community are influenced by the available organic matter and nutrients in the EPS (Louati et al. 2015).

In some toxic bacteria, such as *Microcystis* spp., the bacteria present in the phycosphere were shown to differ markedly from free-living planktonic ones (Wu et al. 2019) and allow the bacteria the access to specific compounds, such as vitamins and some components of the outer membrane lipopolysaccharide, while providing bacteria with highly bioavailable carbon. For example, *Microcystis* spp. do not have the ability to fix nitrogen and appeared to benefit from the nitrogen released from the *Rhizobiales* present in their microbiome and to the enrichment of N_2_-fixation genes (Jankowiak and Gobler 2020).

Raphidiopsis (Cylindrospermopsis) raciborskii (Order Nostocales) is a cyanobacterium that forms toxic blooms that has attracted worldwide interest due to its increasing expansion from warm latitudes to temperate zones. This species is capable of alternately producing saxitoxins (STX) or cylindrospermopsin (CYN) or being non-toxic. However, strains capable of synthesising both STX and CYN at the same time have not yet been described (Vico et al. 2020, Soares et al. 2012, Burford et al. 2018, Antunes et al. 2015, Neilan et al. 2002, Briand et al. 2004, Moreira et al. 2014, Wood et al. 2014, Dokulil 2015, Aguilera et al. 2019).

Although it is proposed that the success of this species in tolerating, colonising and adapting to different environmental conditions (temperature, light and nutrients) is due to a strategy combining phenotypic plasticity (Bonilla et al. 2016, Bonilla et al. 2012, Willis et al. 2018, Soares et al. 2012) and the existence of different ecotypes (Piccini et al. 2011), it is unknown how these environmental changes affect the structure of the microbiota. The increasing incidence of toxic blooms of saxitoxin-producing (SxP) *R.raciborskii* in South America raises concern, since this alkaloid has a very serious neurological effect in humans and animals. Therefore, knowing the composition of its microbiome is relevant, not only to understand its role in *R.raciborskii* growth and population dynamics, but also as a means of discovering bacterial taxa able to degrade saxitoxins to use as a water treatment strategy.

This is the first description of the heterotrophic bacterial community associated with *R.raciborskii* strains described as different ecotypes of the species (Vico et al. 2020, Piccini et al. 2011). We used 16S rRNA gene metagenomics to assess the bacterial microbiomes associated with the phycosphere of two *R.raciborskii* strains, one SxP isolated from South America (MVCC19) and one non-producer (NoP) (LB2897) isolated from North America. The dataset describes the amplicon sequence variants (ASVs) associated with each strain and their taxonomic affiliations.

## Methods

### Cyanobacterial strains, culturing conditions and samples

We analysed the microbiome of two strains of *R.raciborskii* from the Americas, one isolated from a lake located at the northernmost latitude where the species was detected and the other from a lake at the southernmost latitude where the species can be found. The LB2897 strain, originally isolated from Lemon Lake (USA, 39.2568, -86.3929) was obtained from the UTEX culture collection (see Yilmaz and Phlips 2011 for further description) and the MVCC19 strain was isolated in 2007 from Javier Lake (Uruguay, -34.8640, -56.0409) (Vidal and Kruk 2008, Piccini et al. 2011) (Table 1).

Static cultures of both strains were grown in a nitrogen-free BG11 medium (Stanier et al. 1971) at 26°C and PAR light intensity of 80 μmol photons m^2^s^−1^ with a 16:8 h light:dark photoperiod. Under these conditions, the morphology of the two strains was analysed and compared, measuring 30 cells and 50 filaments under the microscope (400 x and 1000 x magnifications). The biovolume was calculated following Hillebrand et al. 1999. The genome of both strains and their phylogenetic and ecological characteristics have been previously published (Vico et al. 2020). The MVCC19 and LB2897 whole-genome shotgun project has been deposited at DDBJ/ENA/GenBank under the accession VIRO00000000 and VOIM00000000, respectively (Vico et al. 2020). After 7 days of incubation in the above-mentioned conditions, 5 ml of each strain culture were sampled and subjected to DNA extraction.

### DNA extraction and 16S RNA gene sequencing

To harvest the cells, samples were filtered on to 2 μm pore size polycarbonate hydrophilic membranes. The DNA extraction was performed as described in Martínez de la Escalera et al. 2014 using the filters containing the biomass as starting material. Briefly, the lysis was achieved by incubating the filters in extraction buffer containing 1% CTAB, EDTA and proteinase K at 37°C during 30 min on a shaker. Then, SDS was added and the mix was incubated at 65°C for 2 h. The resulting lysate was separated from the proteinaceous phase by centrifugation using 24:1 chloroform:isoamylalcohol (three times). After collecting the aqueous upper phase, DNA was precipitated with 0.1 vol. sodium acetate, pH 5.2 and 0.6 vol. isopropanol for 1 h at room temperature. Precipitated DNA was pelleted by centrifugation at 12,000 x g, 45 min, washed with 70% (v/v) cold ethanol (same centrifugation procedure) and suspended in 1 x TE overnight at 4°C. After extraction, the concentration and purity of DNA were spectrophotometrically determined at 260 and 280 nm (NanoDrop).

The 16S rRNA gene was amplified (three pooled technical replicates) and sequencing at the Macrogen Sequencing Service (South Korea). Sequencing libraries were prepared according to the Illumina 16S Metagenomic Sequencing Library protocols to amplify the V3-V4. Primer sequences used for the first amplifications were as follows: 341F/805R(V3-V4, 341F: CCTACGGGNGGCWGCAG, 805R: GACTACHVGGGTATCTAATCC) (Herlemann et al. 2011). The final purified product was then quantified using qPCR according to the qPCR Quantification Protocol Guide (KAPA Library Quantification Kits for Illumina Sequencing platforms) and qualified using the TapeStation DNA screentape (Agilent Technologies, Waldbronn, Germany). Then, the paired-end (2 × 300 bp) sequencing was performed by the Macrogen Sequencing Service, using the MiSeq^TM^ platform.

### Data processing

Bioinformatic analyses of the microbiome were performed in R (version 4.1.0) using the DADA2 package (Callahan et al. 2016). Quality profiles of the forward and reverse reads were inspected by the recommended parameters of DADA2. Sequences were then quality filtered, denoised, merged and the chimera were removed using the DADA2 and amplicon sequence variants (ASVs) of the V3-V4 region of 16S rRNA gene were defined and taxonomically classified using version 132 of Silva Database as a reference (Quast et al. 2012, Glöckner et al. 2017). For multiple alignments, the Decipher package was used (Wright 2015) and the phylogeny tree was constructed with Phangorn (Schliep 2010) with the parameters recommended by Callahan et al. 2016 (see Suppl. material 1)

The composition of bacterial microbiomes at different taxonomic levels was analysed with the Phyloseq package (McMurdie and Holmes 2013) and plots were generated using the package Ggplot2 (Gómez-Rubio 2017). The sample rarefaction analyses were conducted using the library Ranacapa (Kandlikar et al. 2018) (Suppl. material 2). Alpha-diversity was assessed as the Shannon Diversity Index and beta-diversity as the Bray–Curtis distance. ASVs abundances were normalised by proportion (counts in each sample/column were scaled by the sample/column's sum).

## Biodiversity scope

This study was focused on the microbiome of the bloom-forming cyanobacterium *Raphidiopsisraciborskii*, analysing the differences between the microbiome community composition of a saxitoxin-producing strain and a non-toxic one.

### Target

16S ribosomal ARN gene.

### Taxonomic range

Bacterial domain.

## Data Resources

Sequence data from this study have been deposited to NCBI SRA database. Resource identifiers are PRJNA737279 for the taxa obtained from *R.raciborskii* MVCC19 microbiome and PRJNA737280 for those obtained from *R.raciborskii* LB2897.

## Taxonomic composition of the microbiome

**Figure 1.**
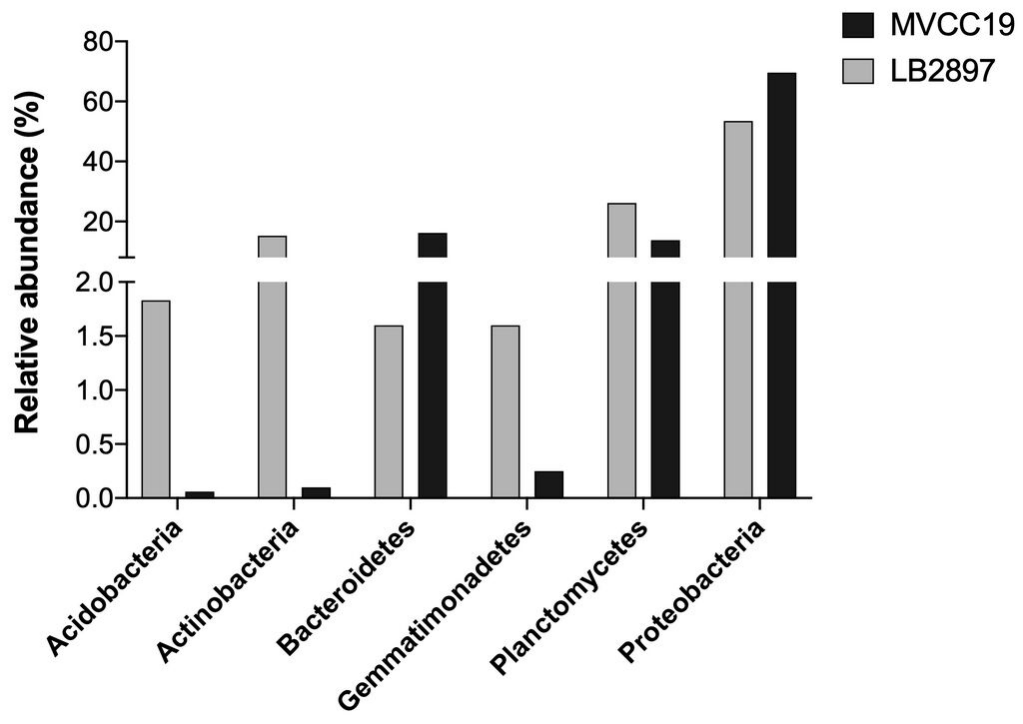
Relative abundance of bacterial phyla found in *R.raciborskii* microbiome from the SxP ecotype (MVCC19, black bars) and NoP ecotype (LB2897, grey bars).

After filtering by quality, denoising, merging and removing the chimeras, a total of 50,753 and 35,908 reads were obtained for the microbiome of LB2897 and MVCC19, respectively. They were clustered into amplicon sequence variants (ASVs) with 100% sequence identity. Taxa richness was 31 and 22 and Shannon Diversity was 2.2 and 2.4 for LB2897 and MVCC19, respectively. Bray–Curtis Distance Index between both microbiomes was 0.26. The most represented phylum was Proteobacteria. Amongst these, the Alphaproteobacteria were dominant (Table 2, Fig. 1).

In both microbiomes, few bacterial genera accounted for more than 50% of the community. In the case of the SxP (*R.raciborskii* MVCC19), the most abundant bacteria belonged to the *Rhizobium*–*Allorhizobium*–*Agrobacterium* clade (or *Neorhizobium*) (Mousavi et al. 2014) (Rhizobiaceae family, Alphaproteobacteria class), with a relative abundance of 22.5%, followed by 13.8% *SM1A02* (Phycisphaeraceae family, Planctomycetes), 12.3% *Brevundimonas* (Caulobacteraceae family, Alphaproteobacteria class) and 9.8% *Emticicia* (family Cytophagaceae, phylum Bacteroidetes).

**Figure 2.**
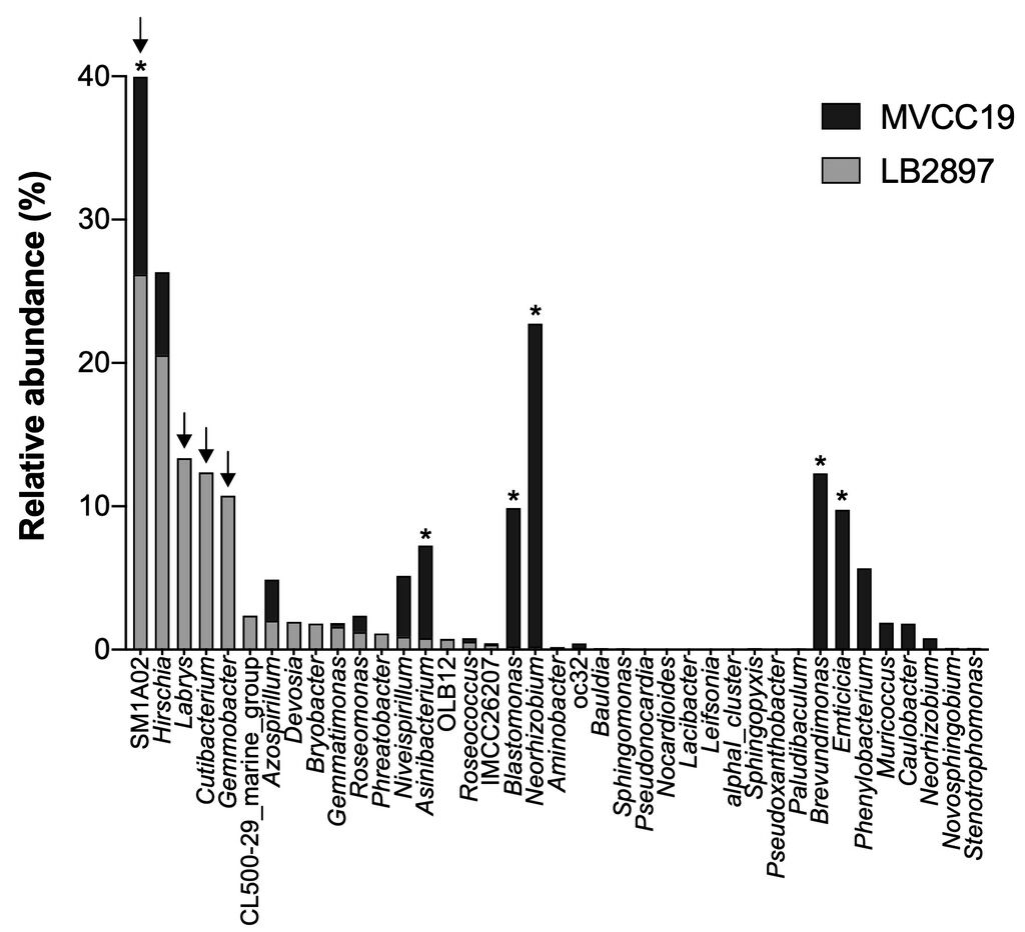
Abundance of the bacterial genera found in the microbiome of SxP (toxic MVCC19) and NoP (non-toxic LB2897) ecotypes of *R.raciborskii*. Asterisks and arrows indicate those genera accounting for more than 50% of the community in the SxP ecotype (MVCC19, black bars) and NoP ecotype (LB2897, grey bars), respectively.

In the NoP (*R.raciborskii* LB2897), *SM1A02* was the most abundant genus (26.2% of the total), followed by 20.5% *Hirschia* (Hyphomonadaceae family, Alphaproteobacteria class), 13.3% *Labrys* (Xanthobacteraceae family, Alphaproteobacteria class) and 12.36% *Cutibacterium* (Propionibacteriaceae family, Actinobacteria class) (Fig. 2).

Taxonomic composition of the microbiome

### Discussion

The data, presented in this work, show that the composition of the bacterial community inhabiting the EPS of two strains of *R.raciborskii* is different, mainly due to the dominant genera. In the case of the NoP ecotype, the 16S rRNA sequences of the most abundant taxa share high identity with bacteria from wastewater treatments and sediment (Table 2). Wastewater bacteria are characterised by being very efficient in nutrient removal (Villaverde 2004). This seems to be the case of the *SM1A02* genus, dominant in NoP microbiome, but also abundant in the phycosphere of SxP. It has been proposed as a novel anammox (anaerobic ammonia oxidiser) bacterium (Tian et al. 2017) found in many activated sludges with good nitrifying performance (Chen et al. 2019). It has been also found in the phycosphere of the marine microaglae *Gambierdiscus* from different locations (Pacific Ocean, Atlantic Ocean, Caribbean Sea), suggesting that it may be broadly associated with this dinoflagellate (Rambo et al. 2019). The anaerobic oxidation of ammonium to nitrogen gas appears to be a metabolic pathway present in virtually any anoxic environment where fixed nitrogen (ammonium, nitrate, nitrite) is found. Therefore, in order to thrive in the EPS layer of *R.raciborskii*, the *SM1A02* anammox bacteria should be located in the anaerobic niches generated by degradation of the mucilage.

In the SxP ecotype, a genus affiliated to *Neorhizobium* (absent in NoP), was the most abundant taxon (Table 2). Bacteria from the Rhizobiaceae family are well known as symbiotic, nitrogen-fixing organisms that live in close association with plants. This nitrogen-fixing redundance found in the toxic strain could reflect the fact that the saxitoxin molecule is nitrogen-rich, probably requiring high concentration of this nutrient to be produced under the nitrogen-deprived conditions of the BG11 medium. Due to the extreme oxygen sensitivity of nitrogenase, the environmental oxygen partial pressure regulates the nitrogen fixation activity. Thus, it is a process that requires anaerobic or microaerobic conditions. In the case of rhizobia, a finely-tuned symbiosis with a plant is the most common strategy, which leads to a root nodule that avoids the high oxygen concentrations generated during photosynthesis (Ledermann et al. 2021). However, as our data do not provide functional information, more work involving, for example, RNA sequencing should be performed in order to determine the spatial location of active *Neorhizobium* inside the EPS of a cyanobacterium, as well as the actual role of this highly abundant microorganism in *R.raciborskii* EPS.

Other abundant bacterial groups associated to the SxP strain have been found in environments contaminated with polycyclic aromatic hydrocarbons (Yang et al. 2018) or nanoparticles (Jomini et al. 2015). The presence of highly abundant bacteria (from 5 to 9%) with the ability to cope with complex compounds could be associated with the use of saxitoxin and analogues as carbon and nutrient source. In addition to organisms related to the nitrogen cycle, we also found taxa commonly found in water or sediment, cyanobacterial blooms and associated to eukaryotic microorganisms, these latter having a symbiotic lifestyle (Table 2).

Hence, the microbiome community, inhabiting the phycosphere of *R.raciborskii* grown under nitrogen-free conditions, showed a combination of functional groups involved in nitrogen cycling, degradation of complex organic compounds and the presence of symbiotic organisms. This functional coupling taking place in the phycosphere of *R.raciborskii* could be related to its small genome content (Stucken et al. 2010). This genome reduction decreases the replication-related energy costs and can be overcome by obtaining metabolic products from the community (Cook et al. 2019). In this way, shifts in the microbiome may help the bacteria to cope with changing environmental conditions faster than by mutation and selection, implying that the whole community represents the unit of natural selection (Rosenberg et al. 2007).

Moreover, some of the taxa, accounting for the higher bacterial richness observed in the NoP microbiome, showed relative abundances ranging from 0.1 to 12.4% and were affiliated to bacteria described as symbionts of eukaryotic organisms (aquatic plants and mosses, ciliates, amphipods) (Table 2). The abscence of these bacteria in the microbiome of the SxP ecotype probably reflects not only the differences in toxin production, but also the different environmental characteristics of the lakes from where they were isolated (Table 1) and suggests a tighter coupling between the non-toxic cyanobacterium and its heterotrophic partners. Since bacterial ecotypes are characterised by having different specific niches and responses to the environment (Cohan 2019), our results suggest that the mechanisms involved in the assembly of the microbiome community are ecotype-dependent. This implies that, to understand and predict the appearance and dominance of bacteria in different environmental conditions, it would be necessary to study them as a community of organisms.

### Conclusions

We found that the dominant bacterial genus in SxP and NoP microbiomes is involved in nitrogen metabolism. Interestingly, while the *SM1A02* genus reached its highest abundance in NoP, it also showed a high relative abundance in SxP. On the other hand, the most abundant genus in SxP (*Neorhizobium*) was in extremely low abundance in NoP. *SM1A02* has been described as anaerobic ammonia oxidisers (anammox) that convert ammonium and nitrite to nitrogen gas and *Neorhizobium* (22.5%) is a nitrogen-fixing Alphaproteobacteria. As saxitoxin and analogues are nitrogen-rich molecules (7 atoms per molecule), toxic ecotypes would need more nitrogen supply than non-toxics to maintain fitness. We hypothesise that saxitoxin-producing *R.raciborskii* benefits from external sources of nitrogen provided by the anammox and nitrogen-fixers partners.

## Caveats and Limitations

The cultures of both strains were performed without added nitrogen, which implies that nitrogen fixation is the main way of obtaining the needed reduced nitrogen to growth. Further studies involving different nitrogen concentrations in the culture medium should be performed in order to determine if the dominant members of the microbiome are still those related to nitrogen cycling.

Caveats and Limitations

## Usage Rights

Sequence data from this study will be publicly available at NCBI.

Usage Rights

## Supplementary Material

8F9B07A6-6EDC-5DF2-8BDE-7213684699CB 10.3897/BDJ.9.e72514.suppl1 Supplementary material 1 Sequence ASV table Data type Phylogenetic Brief description The representative sequence of each identified ASV and their taxonomic identity according to SILVA database are shown. File: oo_572950.xls https://binary.pensoft.net/file/572950 Paula Vico, Andrés Iriarte, Claudia Piccini

189ECF2B-5D7F-5BCF-8D84-948087137BB7 10.3897/BDJ.9.e72514.suppl2 Supplementary material 2 Rarefaction curves Data type image Brief description Rarefaction curves obtained from the 16S rRNA gene sequencing for each *R.raciborskii* strain File: oo_598214.tiff https://binary.pensoft.net/file/598214 Paula Vico, Andrés Iriarte, Sylvia Bonilla, Claudia Piccini

## Figures and Tables

**Table 1.** Morphology, toxicity and biogeographic characteristics of *R.raciborskii* MVCC19 and *R.raciborskii* LB2897. K, conductivity. SD, standard deviation. STX, saxitoxin and analogues. CYN, cylindrospermopsin. Number of data is indicated within brackets. ^a^Bonilla et al. 2016, ^b^Clark and Jones 2006,2011,2013, ^c^Soma et al., pers comm., ^d^Piccini et al. 2011, ^e^Vico et al. 2020.

	**MVCC19**	**LB2897**
**Lake where strain was isolated, coordinates**	Lago Javier^a^ (lat, lon). -34.8640, -56.0409	Lemon Lake^b^ (lat, lon) 39.2568, −86.3929
**Origin/water use^a^**	Eutrophic artificial lake/recreation^a^	Artificial lake/recreation^b^
**Climate**	Subtropical^a^	Tempered^b^
**Area (km^2^)**	0.235^a^	6677^b^
**Max depth (m)**	10^a^	6.1^b^
**K (mScm^-1^) ± SD surface/bottom**	0.48 ± 0.02 (6) /0.55 ± 0.02 (6)^c^	0.154 ± 27.54 (18)/0.156 ± 33.1(18)^b^
**pH ± SD**	8.4 ± 0.08 (53)^a^	8.08 ± 1.01 (21)^b^
**Temperature (°C)** **winter/summer**	11/25.8^a^	-5.0/30.0^b^
**Total phosphorus (mgl^-1^) ± SD**	0.083 ± 0.008 (54)^a^	0.077 ± 0.03 (30)^b^
**Morphology**		
**Filament morphology**	Straight and separate	Straight and slightly curved, bonded
**Filament length (µm) ± SD**	130.3 ± 55 (50)	162 ± 84 (50)
**Filament width (µm) ± SD**	2.57 ± 0.4 (50)	2.09 ± 0.26 (50)
**Cell length (µm) ± SD**	8.3 ± 0.4 (30)	8.5 ± 0.6 (30)
**Filament volume (µm^3^)**	590.2 (50)	556.11 (50)
**Heterocyte position**	Terminal	Terminal
**STX production (STX/GTX2/GTX3)**	Yes^d,e^	No^d,e^
**CYN production**	No^d^	No^d^
**Biogeographic characteristics**	South American clade^e^	Ancestral to South American clade and related to Northern Africa strains ^e^

**Table 2.** GenBank retrieved sequences having more than 97% identity with the ASVs obtained in this study.

**Genus**	**Relative abundances (%)**		**Closer relative (% identity ≥ 97) and GeneBank accession number**	**Environmental source**
	LB2897	MVCC19		
*SM1A02*	**26.17**	13.79	Uncultured bacterium partial 16S rRNA gene (99%) LR643748	Wastewater treatment system
*Hirschia*	20.54	5.80	Uncultured Hyphomonadaceae bacterium clone 1d_33690 (100%) MG805085	Sewage sludge of the completely autotrophic nitrogen removal over nitrite process with a submerged aerated biological filter and the effect of inorganic carbon on nitrogen removal and microbial activity.
*Labrys*	13.35	0.00	Uncultured alpha proteobacterium clone cafs1349 (100%) MF438647.1	Floodplain lake water
*Cutibacterium*	12.36	0	More than 100 sequences having 100% identity, including *C.acnes* (MT242489) and environmental clones associated with P*aramecium* (MH556018)	Oral microbiome. Isolation and Characterization of Predominant Microorganisms during decomposition of Waste Materials in a Field-Scale Composter
*Gemmobacter*	10.74	0	*Gemmobacteraquaticus* strain 05SS-25 (100%) MG780340	Freshwater sediment
*Neorhizobium*	0.23	**22.51**	*Rhizobium* sp. TH167 (100%) KT826347	Cyanobacterial aggregates
*Brevundimonas*	0	12.30	*Brevundimonaslenta* strain P4-2 (100%) MN181016, MH348813, MG642117	Water. Constructed wetlands. Ice
*Blastomonas*	0.24	9.64	*Blastomonas* sp. strain MPSM-12 (100%) MG494710	Daphnia is a reservoir for mercury-tolerant bacteria in the environment
*Azospirillum*	2.03	2.86	Uncultured bacterium clone SIP21-RS-6 (99%) FR774694	Rice rhizospheric soil
*Asinibacterium*	0.80	6.47	*Sediminibacterium* sp. strain FW305-C-49 (99%) MK402932	Groundwater
*CL500-29_marine_group*	2.36	0	Uncultured bacterium clone Wat111 (100%) KC189789	Bacterial Community Structure on *Hydrillaverticillata* and *Vallisneriaamericana* in a Freshwater Spring
*Niveispirillum*	0.91	4.23	LT628527.1	Eutrophic lake. associated with cyano-bloom
*Phenylobacterium*	0	5.68	Uncultured bacterium clone SPN0-300day-93 (99%) MF085152	PAHs contaminated soil
*Bryobacter*	1.81	0	Uncultured bacterium clone (100%) LC336249	Down-flow Hanging Sponge (DHS) reactor treating toluene gas as carbon source
*Devosia*	1.94	0	Uncultured bacterium clone (100%) LR640062	Wastewater treatment system
*Emticicia*	0.00	9.76	Uncultured bacterium clone PlExp_89 (97%) KJ818846	Impact of manufactured TiO nanoparticles on planktonic and sessile bacterial communities (Moselle river)
*Gemmatimonas*	1.60	0.25	Uncultured *Aquabacterium* sp. clone bac21-T3-T2 (100%) KY606809	Hot water biofilm after heat shock treatment
*Roseomonas*	1.23	1.14	*Roseomonas* sp. strain FW305-C-119 (100%) MK402959	Groundwater
*Phreatobacter*	1.12	0	Uncultured *Rhizobiales* bacterium clone 1d_92826 (100%) MG803495	Sewage sludge of the completely autotrophic nitrogen removal over nitrite process with a submerged aerated biological filter and the effect of inorganic carbon on nitrogen removal and microbial activity.
*OLB12*	0.75	0	Uncultured bacterium clone F5K2Q4C04I6QGN (99%) GU911896	Activated sludge
*Muricoccus*	0	1.88	*Roseomonas* sp. strain FW305-C-119 (99%) MK402959	Groundwater
*Caulobacter*	0	1.82	Uncultured bacterium clone HK31-1-39-10 (100%) KX163332	Basaltic subsurface ecosystems
*Roseococcus*	0.55	0.25	Uncultured bacterium clone N3 (100%) HQ697534	Biologically activated carbon for drinking water treatment
*IMCC26207*	0.36	0.10	Uncultured bacterium clone SZB6 (100%) AM176889	Mangrove sediment
*Neorhizobium*	0	0.80	*Rhizobium* sp. strain A&R_E177 (97%) KX550303	Chimney Hills Pond, Tulsa
*Aminobacter*	0.18	0.00	Uncultured bacterium (100%) LR654214	Wastewater treatment system
*oc32*	0.15	0.29	Betaproteobacteria bacterium *5-B6* (98%) LC523959	Root of aquatic plant
*Bauldia*	0.11	0	Uncultured bacterium clone MPB2-18 (99%) AB630694	Microflorae of aquatic moss pillars in a freshwater lake, East Antarctica
*Sphingomonas*	0.09	0	*Sphingomonas* sp. strain SM1-b (100%) MT279454	Exopolysaccharide-producing bacteria from the Ghadikola lagoon water.
*Pseudonocardia*	0.07	0	*Pseudonocardia* sp. strain IB2014P10-1 (100%) MH978626	Actinobacteria associated with deep-water endemic amphipods of Lake Baikal
*Nocardioides*	0.07	0	*Nocardioideskribbensis* strain P86 (100%) MT487642	Spacecraft associated microbial organisms from the Mars odyssey and Pathfinder missions
*Sphingopyxis*	0.04	0.08	*Sphingopyxis* sp. strain T93 9100%) MT611302	Bacteria isolated from highland barley cultivation soil in Tibet
*Lacibacter*	0.06	0	Uncultured prokaryote clone OTU029 (99%) KF680692	Drinking water biofilm
*Leifsonia*	0.05	0	Uncultured Microbacteriaceae bacterium clone UVmen1_31 (99%) JQ701147	Water from long-term experimental oligotrophic mesocosms in Cuatro Cienegas"
*alphaI_cluster*	0.05	0	Uncultured bacterium clone Espejo_1_17_12_Water.240996 (98%) KM184952	Water from Espejo lake, Argentina
*Paludibaculum*	0.03	0.06	Uncultured bacterium clone FL_03_167 (100%) KC666531	Bacterial communities associated to *Microcystis* colonies
*Novosphingobium*	0	0.14	Uncultured bacterium clone LNH_9_9_11_Pumice.207741 (99%) KM124853	Water from Nahuel Huapi lake, Argentina
*Stenotrophomonas*	0	0.14	No match	-
*Pseudoxanthobacter*	0.04	0	Uncultured bacterium clone EF_bacC09 (100%) JX564275	Sediment from slow sand filtration columns (wastewater)
