# Supplementary figures and images for: Metagenomic analysis of Raphidiopsisraciborskii microbiome: beyond the individual
Source: Biodivers Data J. 2021 Oct 21;9:e72514. doi: 10.3897/BDJ.9.e72514 (PMC8553701; doi:10.3897/BDJ.9.e72514)

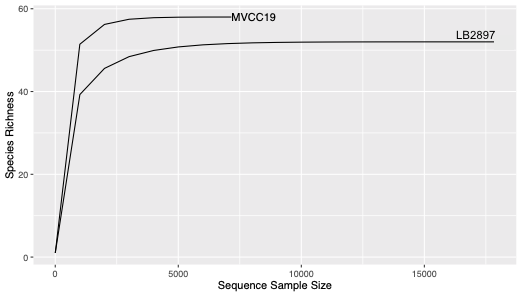

Supplement: Supplementary material 2 — Rarefaction curves [file bdj-09-e72514-s002.tiff]
